# Altered Aconitase 2 Activity in Huntington’s Disease Peripheral Blood Cells and Mouse Model Striatum

**DOI:** 10.3390/ijms18112480

**Published:** 2017-11-21

**Authors:** Chiung-Mei Chen, Yih-Ru Wu, Kuo-Hsuan Chang

**Affiliations:** Department of Neurology, Chang Gung Memorial Hospital, Linkou; Chang-Gung University College of Medicine, Taoyuan 33305, Taiwan; yihruwu@cloud.cgmh.org.tw (Y.-R.W.); gophy5128@gmail.com (K.-H.C.)

**Keywords:** Huntington’s disease, Hdh^(CAG)150^ and R6/2 mice, peripheral blood mononuclear cells, aconitase 2, *N*-acetyl-l-cysteine, biomarker

## Abstract

Huntington’s disease (HD) is caused by an unstable cytosine adenine guanine (CAG) trinucleotide repeat expansion encoding a polyglutamine tract in the huntingtin protein. Previously, we identified several up- and down-regulated protein molecules in the striatum of the Hdh^(CAG)150^ knock-in mice at 16 months of age, a mouse model which is modeling the early human HD stage. Among those molecules, aconitase 2 (Aco2) located in the mitochondrial matrix is involved in the energy generation and susceptible to increased oxidative stress that would lead to inactivation of Aco2 activity. In this study, we demonstrate decreased Aco2 protein level and activity in the brain of both Hdh^(CAG)150^ and R6/2 mice. Aco2 activity was decreased in striatum of Hdh^(CAG)150^ mice at 16 months of age as well as R6/2 mice at 7 to 13 weeks of age. Aco2 activity in the striatum of R6/2 mice could be restored by the anti-oxidant, *N*-acetyl-l-cysteine, supporting that decreased Aco2 activity in HD is probably caused by increased oxidative damage. Decreased Aco2 activity was further found in the peripheral blood mononuclear cells (PBMC) of both HD patients and pre-symptomatic HD mutation (PreHD) carriers, while the decreased Aco2 protein level of PBMC was only present in HD patients. Aco2 activity correlated significantly with motor score, independence scale, and functional capacity of the Unified Huntington’s Disease Rating Scale as well as disease duration. Our study provides a potential biomarker to assess the disease status of HD patients and PreHD carriers.

## 1. Introduction

Huntington’s disease (HD), an autosomal-dominant neurodegenerative disorder, is mainly characterized by different psychiatric symptoms, progressive mental decline and chorea. The genetic mutation of HD is an expanded cytosine adenine guanine (CAG) trinucleotide repeat that encodes a polyglutamine (polyQ) tract in the huntingtin (Htt) protein [1]. The polyQ expansion causes a conformational change in the Htt which tends to form intranuclear and cytoplasmic aggregates of affected neurons, which would lead to progressive neuronal dysfunctions [2,3]. Impaired proteasome activity [4], transcriptional dysregulation [5], oxidative stress [6], mitochondrial and metabolic dysfunction [7], abnormal protein-protein interaction [8], neuroinflammation [9,10], and microglial activation [11,12,13,14] have been shown to play important roles in the pathogenesis of HD. While many pathogenic pathways have been uncovered, therapeutic strategies that prevent HD progression or modify disease course are not available yet. Many potential treatments have been reported in cell and/or animal models, but none of them proves to be effective in clinical trials. One of the major drawbacks of the clinical trial for HD treatments is the lack of a useful biomarker that can be used to test the therapeutic efficacy.

Several mouse models of HD have been established not only to explore the pathogenic mechanisms but also to test the potential therapeutic strategies [15,16,17]. These models include the R6/2 transgenic mice expressing *exon* 1 of the human *Htt* gene carrying 150 CAG repeats [15] and knock-in mouse models manifesting mild phenotypes with late disease onset and late occurrence of intracellular aggregates without prominent neuronal loss, indicating that knock-in mice are modeling early phase of human HD [16,17,18]. The knock-in mice are therefore useful tools for investigating early pathological events in HD. Usually, a significant finding in one mouse model needs to be also shown in another model to confirm the result.

Previously, by investigating the proteome profile in the striatum of the Hdh^(CAG)150^ knock-in mice [16] at 16 months of age when most heterozygous Hdh^(CAG)150^ mice manifested phenotype, we identified several up- and down-regulated protein molecules (Supplementary materials and methods, Figure S1 and Table S1). Among those differentially-expressed molecules, the aconitase 2 (Aco2) activity was also found to be decreased in the striatum of R6/2 HD mice at late stage [19] and postmortem brains of HD patients [20,21]. Chiang and colleagues also found decreased Aco2 protein in striatum of HD R6/2 mice at 12 weeks of age [22]. Aco2 located in the mitochondrial matrix is an iron-sulfur protein that requires a 4Fe-4S cluster for its enzymatic activity and its function is to catalyze conversion of citrate to isocitrate in the tricarboxylic acid (TCA) cycle, an important step involved in the ATP generation. Aco2 is susceptible to increased oxidative stress that would inactivate Aco2 activity [20]. While reduced Aco2 activity in the late stage of R6/2 mice has been reported [19], there are no studies to examine if loss of Aco2 is an early and progressive change in central nervous system and peripheral blood of HD patients. Since mitochondrial abnormalities and increased oxidative stress have been suggested to play an important role in pathogenesis of HD, we investigated if decreased Aco2 is present in early disease stage and if it progresses with time in Hdh^(CAG)150^ and R6/2 HD mice. We then examined the effect of an anti-oxidant, *N*-acetyl-l-cysteine (NAC), on motor performance and Aco2 activity in striatum of HD mice, and quantified the Aco2 level and activity in peripheral blood mononuclear cells (PBMC) of HD patients and pre-symptomatic HD mutation (PreHD) carriers. Finally, we examined if Aco2 activity in PBMC correlates with Unified Huntington’s Disease Rating Scale (UHDRS) [23] in HD patients and PreHD carriers.

## 2. Results

### 2.1. Rotarod Performance of Hdh^(CAG)150^ and R6/2 Mice

**Figure 1:**
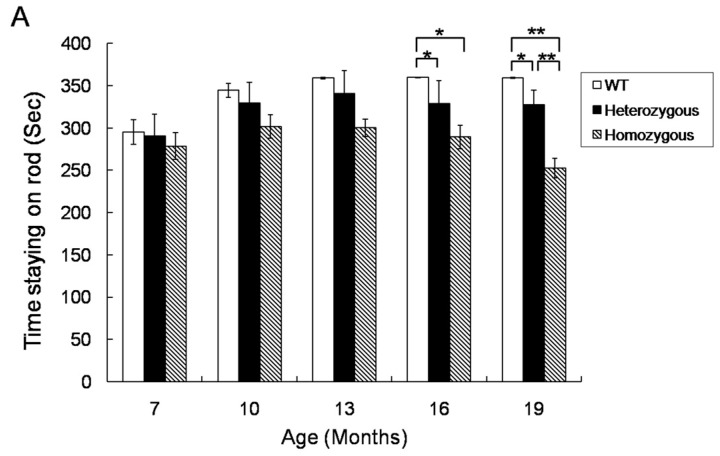

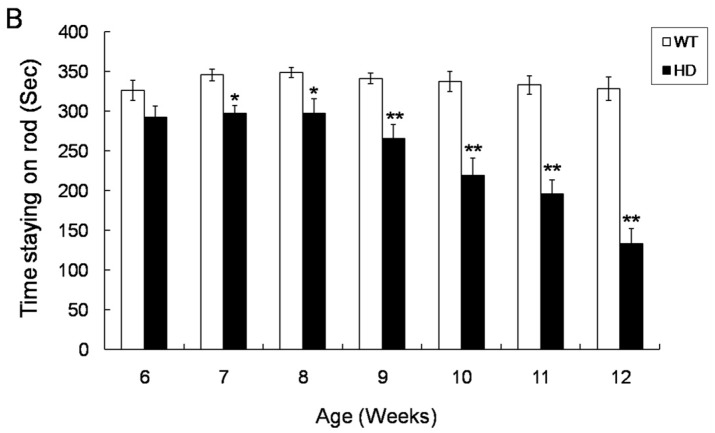
Rotarod performance of Hdh^(CAG)150^ and R6/2 mice. (**A**) Rotarod performances of heterozygous (*n* = 15) and homozygous (*n* = 8) Hdh^(CAG)150^ mice were compared with those of the wild type (WT) littermates (*n* = 15) at 7~19 months of age and (**B**) Rotarod performances of R6/2 mice (*n* = 15) at 6~12 weeks of age were compared with those of the WT littermates (*n* = 15). Latency to falling was automatically recorded. Data are presented as means ± SE (standard error). * *p* < 0.05; ** *p* < 0.01, two-way analysis of variance (ANOVA) with post-hoc Tukey test.

In order to examine the correlation between the Aco2 level and the HD phenotype, the motor performance of Hdh^(CAG)150^ and R6/2 mice was tested by using a rotarod device. The rotarod performance of heterozygous and homozygous Hdh^(CAG)150^ mice at 16 and 19 months of age was significantly worse than that of their wild type (WT) littermates, respectively (Figure 1A). The rotarod performance of homozygous Hdh^(CAG)150^ mice was worse than that of heterozygous mice at 19 months of age. The rotarod performance of R6/2 mice was significantly worse compared with their WT littermates at 6 to 12 weeks of age (Figure 1B).

### 2.2. Decreased Aco2 in the Striatum of Hdh^(CAG)150^ Mice at 16 Months of Age

**Figure 2:**
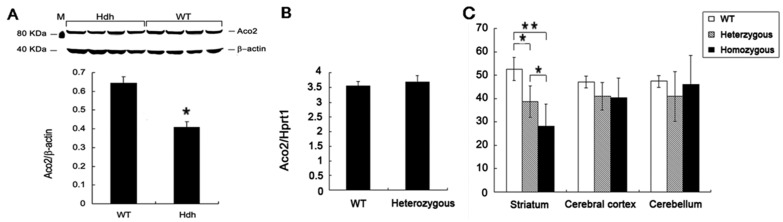
Aconitase (Aco2) expression and activity in the brain regions of Hdh^(CAG)150^ (Hdh). (**A**) Western blot analysis of Aco2 in the striatum of heterozygous Hdh^(CAG)150^ (Hdh) mice and the wild type (WT) littermates at 16 months of age. M, marker for protein molecular weight; (**B**) *Aco2* mRNA expression level in the striatum of Hdh^(CAG)150^ mice (heterozygous, *n* = 8) compared with that of WT littermates (*n* = 8) at 16 months of age. Expression ratios are relative to *Hprt1* and (**C**) Aco2 activity in the brain regions of Hdh^(CAG)150^ mice. Aco2 activities in the striatum, cerebral cortex, and cerebellum of heterozygous (*n* = 6) and homozygous (*n* = 4) mice were compared with those of their wild type (WT) littermates (*n* = 6) at 16 months of age. Data are presented as means ± SE (standard error bars). * *p* < 0.05; ** *p* < 0.01, two-tailed Student’s *t*-test or one way ANOVA with post-hoc Tukey honestly significant difference (HSD) test, where appropriate.

Protein lysates from brain tissues of heterozygous Hdh^(CAG)150^ and WT littermates at 16 months of age were subjected to western blot for semi-quantification of Aco2. Aco2 protein level was significantly decreased in the striatum of male Hdh^(CAG)150^ mice at 16 months of age (Figure 2A), but such decrease was not seen in the cerebellum and cortex of Hdh^(CAG)150^ mice at the same age (Figure S2). Quantitative real-time PCR (QRT-PCR) using *Aco2*-specific probe and primers showed no significant difference in *Aco2* mRNA expression in striatum between Hdh^(CAG)150^ and WT littermates at 16 months of age (Figure 2B). Aco2 is susceptible to increased oxidative stress that leads to inactivation of Aco2 activity [20]. Therefore, Aco2 activity from mitochondria was measured for Hdh^(CAG)150^ mice at 16 months of age. Aco2 activity was significantly decreased in the striatum, but not in the cerebral cortex and cerebellum, of heterozygous and homozygous Hdh^(CAG)150^ mice at 16 months of age compared with the WT littermates, respectively (Figure 2C). It is also noted that the Aco2 activity was lower in homozygous Hdh^(CAG)150^ mice compared with the heterozygous mice. However, decreased Aco2 activities in different brain regions were not observed in heterozygous and homozygous Hdh^(CAG)150^ mice at 13 months of age (Figure S3).

### 2.3. Decreased Aco2 in Brain Regions of R6/2 Mice from 7 to 13 Weeks of Age

**Figure 3:**
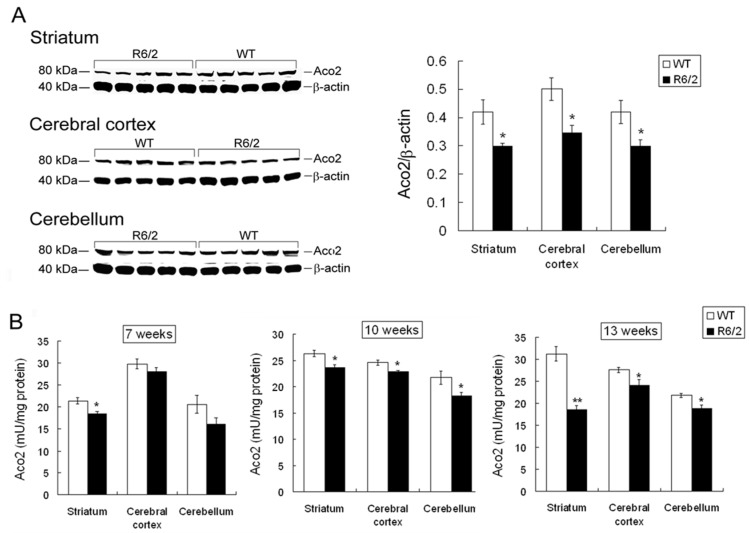
Aco2 protein expression and activity in the brain regions of R6/2 mice. (**A**) Aco2 expression levels, analyzed by western blot, in the striatum, cerebral cortex, and cerebellum of R6/2 mice (*n* = 5) were compared with their wild type (WT) littermates (*n* = 5) at 13 weeks of age. The expression levels of Aco2 were normalized by β-actin and (**B**) Aco2 activities in the striatum, cerebral cortex, and cerebellum of R6/2 mice (*n* = 6) were compared with those of WT littermates (*n* = 6) at 7, 10 and 13 weeks of age. Data are presented as means ± SE (standard error bars). * *p* < 0.05; ** *p* < 0.001, two-tailed Student’s *t*-test.

In order to examine if the decreased Aco2 protein and activity are common pathological events shared by other HD mouse models, Aco2 level and activity in brain regions of R6/2 mice was examined. Aco2 protein level was significantly decreased in the striatum, cortex, and cerebellum of R6/2 mice at 13 weeks (Figure 3A) but not at 10 weeks of age (Figure S4). Aco2 activity in the striatum, cerebral cortex, and cerebellum of R6/2 mice at 10 and 13 weeks of age was significantly decreased compared with the WT littermates (Figure 3B), while this change was found only in striatum and not in cortex or cerebellum of R6/2 mice at 7 weeks of age (Figure 3B). It has been shown that Aco2 is one of the substrates of transglutaminase 2. In order to delineate if decreased Aco2 activity is associated with increased transglutaminase 2 in R6/2 mice, transglutaminase 2 was semi-quantified by using western blot. The result showed that transglutaminase 2 expression levels in the striatum of R6/2 mice from 9 to 13 weeks of age were not different from those in WT littermates (Figure S5).

### 2.4. Aco2 Activity in the Striatum of R6/2 Mice Was Restored by Treatment with NAC

**Figure 4:**
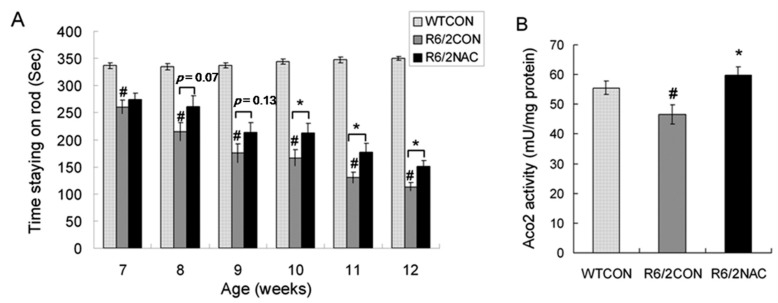
Effect of *N*-acetyl-l-cysteine (NAC) treatment on rotarod performance and Aco2 activity in the striatum of R6/2 mice. (**A**) Rotarod performance of R6/2 mice intraperitoneally injected with NAC (R6/2NAC, *n* = 18) at 7, 8, 9, 10, 11, and 12 weeks of age were compared with those of R6/2 treated with saline (R6/2CON, *n* = 18) and (**B**) Aco2 activities in the striatum of R6/2 mice intraperitoneally injected with NAC (R6/2NAC, *n* = 6) were compared with those of R6/2 mice treated with saline (R6/2CON, *n* = 6) at 12 weeks of age. Data are presented as means ± SE (standard error bars). Specific comparison between R6/2CON and WT mice is labeled as # (*p* < 0.05, one way ANOVA with post-hoc Tukey test). Specific comparison between R6/2NAC and R6/2CON mice is labeled as * (*p* < 0.05, one way ANOVA with post-hoc Tukey test).

Since decreased Aco2 activity was assumed to result from increased oxidative stress, anti-oxidant NAC may restore the Aco2 activity in HD mice by preventing oxidation and inactivation of Aco2. In order to know if Aco2 activity can be a useful biomarker to test the treatment efficacy, we measured Aco2 activity in the striatum of R6/2 mice treated with and without NAC. R6/2 mouse model was used because it shows early onset of symptoms and fast disease progression, and is widely used for therapy screening [24]. We examined the effect of NAC on the rotarod performance, body weight, blood sugar, Htt aggregates, and striatal Aco2 activity of R6/2 mice. The rotarod performance of R6/2 mice from 7 to 12 weeks of age was significantly worse compared with the WT littermates (Figure 4A). While NAC did not have any beneficial effect on the body weight, blood sugar, and Htt aggregates of R6/2 mice (Figure S6), rotarod performance of R6/2 mice treated with NAC was mildly improved at 8 and 9 weeks of age, and significantly improved at 10, 11, and 12 weeks of age compared with those of R6/2 mice treated with saline only (Figure 4A). Aco2 activity in the striatum of R6/2 mice treated with NAC at 12 weeks of age (59.7 ± 2.9 mU/mg protein) was significantly increased when compared with those treated with saline only (46.6 ± 3.2 mU/mg protein) (Figure 4B).

### 2.5. Aco2 Activity of PBMC Was Decreased in HD Patients and PreHD Carriers

**Figure 5:**
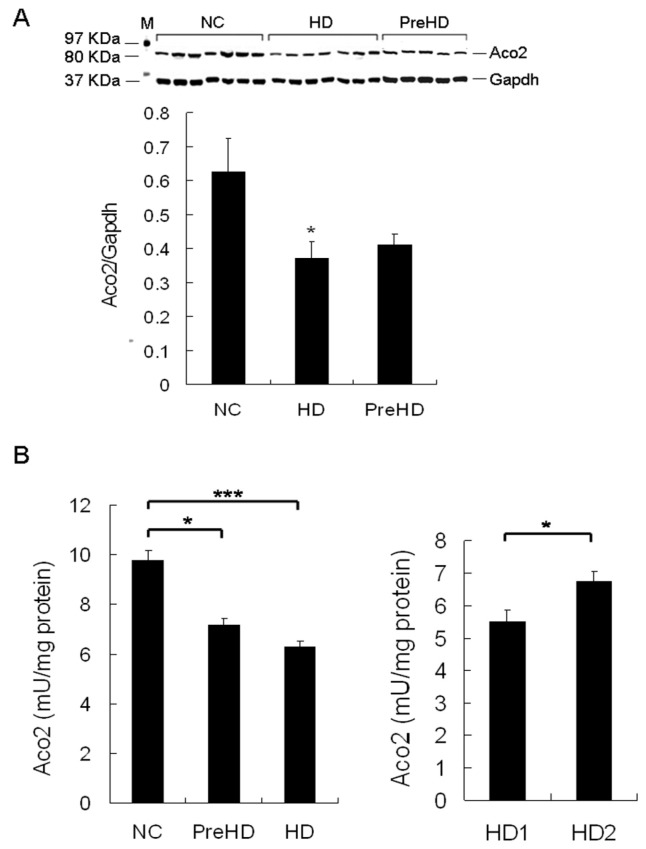
Aco2 protein expression and activity in peripheral blood mononuclear cells (PBMC) of HD patients and pre-symptomatic HD mutation (PreHD) carriers. (**A**) PBMC Aco2 expression levels, analyzed by western blot, in HD patients and PreHD carriers were compared with the control group (NC). The expression levels of Aco2 were normalized by Gapdh. M, marker for protein molecular weight. (**B**) Aco2 activities of PBMC in PreHD carriers (*n* = 6) and HD patients (*n* = 19) were compared with the controls (*n* = 25). HD1 group (*n* = 7): more severe disease group. HD2 group (*n* = 12): milder disease group. Data are presented as means ± SE (standard error). * *p* < 0.05; *** *p* < 0.001, Mann–Whitney–*U* or Kruskal–Wallis test, where appropriate.

**Figure 6:**
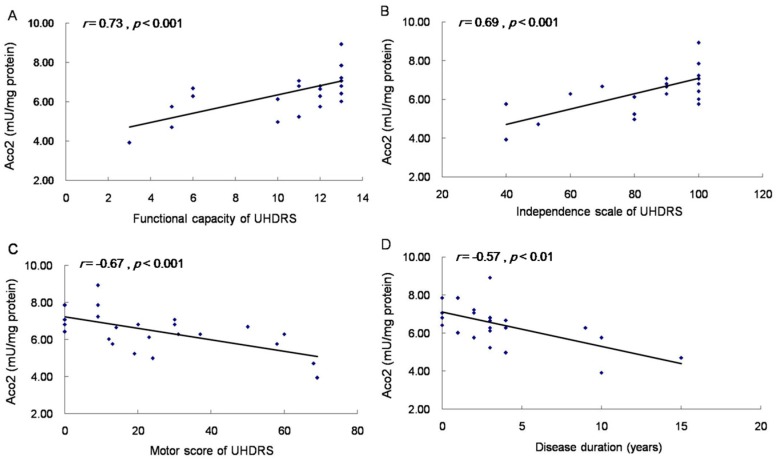
Correlation of Aco2 activity of PBMC with functional capacity, independence scale, and motor score of Unified Huntington’s Disease Rating Scale (UHDRS), and disease duration of HD patients. Spearman correlation showed that Aco2 activity correlated positively with functional capacity (**A**) and independence scale (**B**), and inversely with motor score (**C**) and disease duration (**D**) in 19 HD patients and 6 preHD carriers.

Since our results showed decreased Aco2 protein and activity in brains of different HD mouse models and the alteration in R6/2 mice progressed with time, Aco2 is a good candidate for a biomarker to indicate the disease stage or progression. To get a central nervous system (CNS) sample from HD patients for identifying biomarkers is practically impossible, which makes finding a biomarker in peripheral tissue, especially from blood, more feasible and important. Given that Htt is expressed ubiquitously, and parallel CNS and peripheral pathogenic pathways have been shown [10,25,26,27], we hypothesized that the decreased Aco2 and its activity may be detectable in peripheral blood cells of HD patients and PreHD carriers, and may therefore serve as a potential biomarker. The results showed that Aco2 protein level of PBMC was significantly decreased in HD patients, while not in PreHD carriers (Figure 5A). Aco2 activity of PBMC was significantly reduced in both HD patients (*p* < 0.001, 6.3 ± 0.3 mU/mg protein) and PreHD carriers (*p* < 0.05, 7.2 ± 0.2 mU/mg protein) compared with the controls (9.8 ± 0.4 mU/mg protein) (Figure 5B). Decrease of Aco2 activity was more significant (*p* < 0.05) in HD1 (5.5 ± 0.4 mU/mg protein) than in HD2 group (6.7 ± 0.3 mU/mg protein) (Figure 5B). Aco2 activity correlated positively with independence scale (*r* = 0.69, *p* < 0.001) and functional capacity (*r* = 0.73, *p* < 0.001), and inversely with motor score (*r* = −0.67, *p* < 0.001) and disease duration (*r* = −0.57, *p* < 0.01) (Figure 6). However, Aco2 activity did not correlate with repeat length (*p* = 0.1), age onset (*p* = 0.5), and age (*p* = 0.4) in the HD group or age in the control group (*p* = 0.3).

## 3. Discussion

Decreased Aco2 and its activity were found in Hdh^(CAG)150^ and R6/2 mice in the present study, which provides another line of evidence that mitochondrial abnormalities contribute to neuronal dysfunction in HD. Decreased Aco2 and its activity are present in striatum of Hdh^(CAG)150^ at 16 months of age when the mice began to manifest impaired motor performance. While Aco2 protein level was decreased in striatum, cortex, and cerebellum of R6/2 mice at 13 weeks of age, this change was not found in the brain of R6/2 mice at 10 weeks of age. In contrast, Aco2 activity was reduced in striatum, cortex, and cerebellum of R6/2 mice at 10 as well as 13 weeks of age. The decreased Aco2 activity was further found in the striatum of R6/2 mice at 7 weeks of age when the mice began to show impaired motor performance (7 weeks of age in Figure 1 and Figure 4). Different from our results, a previous study showed increased Aco2 level in R6/2 HD brains at 2 weeks of age, indicating up-regulated energy metabolism, but it is not clear if the Aco2 level was altered in the late disease stages [28]. Although decreased Aco2 activity and protein levels have been respectively shown in the striatum of R6/2 mice in the late disease stage [19,29], the present study demonstrates that decreased Aco2 activity in R6/2 occurred before overt phenotype manifested. The cause of decreased Aco2 activity is not clear, but a couple of hypotheses are proposed. Aco2 is vulnerable to oxidative stress. After oxidative modification by peroxynitrate, Aco2 can lose the enzymatic activity. Aco2 has been found to be the only protein in the mitochondrial matrix that exhibited reduced activity associated with increase in carbonylation under increased oxidative stress condition [30]. Therefore, Aco2 enzyme function is well positioned as an important marker related to biological decline of mitochondria and increased oxidative stress. One explanation of decreased Aco2 in Hdh^(CAG)150^ and R6/2 may be due to increased oxidative damage that has been shown in brain tissues of both HD mouse models and patients [19,20,21,25,31,32,33]. We also have previously shown increased oxidative stress in peripheral blood of HD patients [34]. Increased carbonylated Aco2 in R6/2 mice shown by Perluigi and colleagues further supports this postulation [35]. Another possibility is that decreased Aco2 mRNA expression results in reduced Aco2 activity, which is probably not the case in the present study as no difference in Aco2 mRNA between Hdh^(CAG)150^ and their WT littermates was found (Figure 2C). Furthermore, decreased Aco2 activity occurs before reduced Aco2 protein level in R6/2 mice, suggesting that impaired Aco2 activity was caused by oxidative inactivation followed by degradation rather than by decreased Aco2 transcriptional expression. Increased transglutaminase activity has been found in affected brains of HD patients as well as R6/2 HD mice [36,37,38] and transglutaminase inhibitor (cystamine) improved survival and phenotype of R6/2 HD mice [38]. Interesting work done by Kim et al., has shown that transglutaminase 2 has inhibitory effect on Aco2 activity, contributing to aggregates of Aco2 and that increased transglutaminase activity in the striatum of human HD may be the underlying cause of the reported reduced Aco2 activity in the striatum of HD [39]. Previously, NAC was shown to decrease transglutaminase activity [40], whereas our study did not show increased transglutaminase 2 expression in the striatum of R6/2 mice from 9 to 13 weeks of age, which suggests that the decreased Aco2 activity in R6/2 mice may not result from increased transglutaminase 2 and the NAC effects on R6/2 mice may be not mediated through inhibiting transglutaminase 2.

The reduction of Aco2 in both Hdh^(CAG)150^ and R6/2 mice suggests that decreased Aco2 is a common feature shared by both knock-in and transgenic HD mouse models. As the knock-in Hdh^(CAG)150^ mice model the early stage of HD and decreased Aco2 activity is present before the onset of phenotype of R6/2 mice, we postulated that reduced Aco2 activity may be an early pathogenic event in HD patients. Indeed, this assumption is supported by decreased Aco2 activity of PBMC in PreHD carriers as well as HD patients in our study. Our results are in accordance with the previous repot that in the presence of oxidative stressors, Aco2 activity in lymphoblasts of HD decreased significantly compared with the controls [41]. The decreased Aco2 activity without changes in Aco2 protein level in PBMC of PreHD carriers also implicates that inactivation of Aco2 precedes protein degradation in HD. The reduction is correlated significantly with disease severity indicated by UHDRS as well as disease duration. Therefore, Aco2 activity may serve a potential biomarker for indicating disease status and testing efficacy of future therapeutic strategies. Our study also suggests Aco2 as a potential target of the HD treatment and means to enforce Aco2 activity or decrease oxidative stress will be beneficial to HD patients. This hypothesis is supported by the improved rotarod performance and increased Aco2 activity in the striatum of R6/2 mice treated with the antioxidant NAC in our study. Another line of evidence is that NAC delayed the onset of motor deficits in the R6/1 model of HD by lowering protein carbonylation in mitochondria and enhancing mitochondrial capacity [42]. Our results are also in agreement with the previous findings that several antioxidant agents [6] such as coenzyme Q10 [43,44], BN82451 [45], and triterpenoids [46] are neuroprotective to HD mouse models. Given that antioxidants are emerging as promising treatments for HD, Aco2 activity indicating the degree of oxidative damage may well serve as a surrogate biomarker for clinical trials of potential antioxidants.

Several kinds of abnormalities including increased cytokines, mitochondrial dysfunction, increased oxidative stress, aberrant adenosine A2A receptor function, reduced phosphorylated serine-threonine protein kinase 1 (Akt), increased pro-catabolic serum metabolites, elevated serum 8OH2’dG, deficient fatty acid amide hydrolase activity, energy deficit, and alteration of phosphatidylcholine in peripheral blood of HD patients have been reported [9,10,27,34,47,48,49,50,51,52,53,54,55,56], whereas only a few showed a positive correlation with disease severity or stage [9,10,27,34,51,53,56,57]. Notably, our study has shown that Aco2 activity in the striatum of R6/2 mice could be restored by the anti-oxidant NAC that also improved motor performance of R6/2 mice. Our study provides another potential peripheral biomarker to assess the disease status and progression of HD patients and PreHD carriers. However, there are limitations of our study. Firstly, the main aim of this study is to address if Aco2 is decreased in brains of different HD mouse models and peripheral blood of HD patients, and if Aco2 activity correlates with disease severity to serve as a potential biomarker, but the underlying mechanism is not investigated, which warrants more studies to explore further in the future. Secondly, peripheral Aco2 may not faithfully reflect the pathological changes in brain, although it correlates with disease severity. Molecules in cerebrospinal fluid (CSF) may serve as better biomarkers than those in peripheral blood, in terms of recapitulating the changes in brain. Although it is difficult to get enough CSF samples from our HD patients, in the future we plan to examine the potential targets in CSF of HD patients by getting access to the HD CSF repository (http://hdclarity.net). Future studies to demonstrate simultaneous rescue of Aco2 activity in brain and peripheral blood of R6/2 mouse and other HD animal models by treatment with anti-oxidants are necessary to further consolidate the biomarker role of Aco2 activity in HD. Since our findings are exploratory, confirmation of our results in a larger series, multi-center, and longitudinal evaluation of Aco2 activity in HD patients and preHD carriers is important before applying Aco2 as a biomarker for HD.

## 4. Materials and Methods

### 4.1. Animals and NAC Treatment

The male mice used for the knock-in HD mouse model (Hdh^(CAG)150^, B6.129P2-Hdhtm2Detl/J) were purchased from Jackson Laboratories (Bar Harbor, ME, USA) harboring a mutant mouse *huntingtin* (*Htt*) gene with 150 copies of CAG [16] and mated to female littermates. Offspring were identified by genotyping of tail DNA. PCR genotyping was performed using the following primers: 5′-CCCATTCATTGCCTTGCTGCTAAG-3′ and 5′-GACTCACGGTCGGTGCAGCGGTTCC-3′. Male R6/2 mice [15] were originally obtained from Jackson Laboratories (Bar Harbor, ME, USA) and mated to female control mice (B6CBAFI/J). PCR genotyping was performed using the following primers: 5′-CCG CTC AGG TTC TGC TTT TA-3′ and 5′-GGC TGA GGA AGC TGA GGA G-3′. The tail DNA of F1 progeny of R6/2 mice was sequenced for CAG repeat length. All animals used in this study are male. All animals were housed at the Animal Care Facility, Chang Gung Memorial Hospital and had unlimited access to water and breeding chow (PicoLab^®^ Rodent Diet 20, PMI^®^ Nutrition International, St. Louis, MO, USA) under a 12-h light~12-h dark cycle. Body weights of mice were recorded once daily. R6/2 mice treated with NAC (*n* = 18) or saline (*n* = 18) from 5 to 12 weeks of age were injected intraperitoneally daily with 120 mg/kg NAC dissolved in physiologic saline or with saline only. Animals were anesthetized with sodium pentobarbital and decapitated at the age of 12 weeks. Animal experiments were performed under protocols approved by the Animal Care and Utilization Committee and Institutional Review Board, Chang Gung Memorial Hospital, Taiwan (The project identification code: 2008121803, date of approval: 19 December 2008).

### 4.2. Rotarod Performance

Motor coordination of mice was assessed using a rotarod apparatus (UGO BASILE, Comerio, VA, Italy) at an accelerated speed (4~44 rpm) over a period of 6 min. The animals were pre-trained for one trial at an accelerated speed (4~44 rpm) for 5 min 2 days before the real test to allow them to become acquainted with the rotarod apparatus. Heterozygous (*n* = 15) and homozygous (*n* = 8) Hdh^(CAG)150^ mice and their littermates (*n* = 15) were then tested every 3 months from 7 to 19 months of age. The rotarod test of R6/2 mice (*n* = 15) and the WT littermates (*n* = 15) were performed every week from 6~12 weeks of age. R6/2 mice treated with (*n* = 18) and without NAC (*n* = 18) and the WT littermates (*n* = 18) treated with saline were tested every week from 7 to 12 weeks of age. Each mouse was tested for a maximum of 6 min per trial for 3 trials with an interval of 30 min in a day and mean of the 3 trials was used for comparison between groups. Latency to falling was automatically recorded.

### 4.3. HD Patients and PBMC Preparation

PreHD carriers are defined as individuals carrying CAG repeats longer than 36 with UHDRS motor score ≤ 5, functional capacity at 13, and independence scale at 100. HD patients are defined as individuals carrying CAG repeats longer than 36 with UHDRS motor score > 5. Nineteen HD patients and six PreHD carriers (All were genetically confirmed), and 25 age- and gender-matched healthy individuals without other neurological or major systemic diseases were recruited. Those patients and controls with confounding factors known to influence oxidative stress markers such as taking anti-oxidative or anti-inflammatory compounds were excluded (Table 1). All of them were subjected to Aco2 activity examination. Among them, samples collected from 7 HD patients, 5 PreHD carriers along with the respective groups of age-matched controls were performed for Aco2 western blot. All patients were assessed for UHDRS [23]. The UHDRS is composed of total motor score (0 to 124), independence scale (100 to 10), and total functional capacity (13 to 0). The HD patients were divided into two disease severity groups according to their total functional capacity of the UHDRS (Table 1). Patients with the total functional capacity ≤10 represent the more severe group (HD1). Patients with total functional capacity at 11~13 represent the group of milder disease severity (HD2).

Blood samples were drawn from HD patients, PreHD carriers, and controls using ethylenediaminetetraacetic acid (EDTA)-containing tubes after the participants signing informed consent. Samples were processed for isolation of PBMC immediately after blood collection using Ficoll-Paque™ Plus (GE Healthcare Bio-Sciences, Pittsburgh, PA, USA). The isolated PBMC was further subjected to immunocytochemical staining to detect CD1b, a marker for PBMC (anti-CD1b, 1:200 dilution, Abcam, Cambridge, UK) with nuclei being stained by Hoechst (Figure S7). The human study was performed under a protocol approved by the institutional review boards of Chang Gung Memorial Hospital.

### 4.4. Western Blot Analysis

Heterozygous Hdh^(CAG)150^ mice (*n* = 4) and their littermates (*n* = 4) at 16 months of age, and R6/2 mice (*n* = 5) and the littermates (*n* = 5) at 10 and 13 weeks of age were subjected to western blot analysis of Aco2. R6/2 mice (*n* = 6) and the littermates (*n* = 6) at 9, 11 and 13 weeks of age were subjected to western blot analysis of transglutaminase 2. Equal amounts of protein lysate (25 ìg) from striatum, cortex, or cerebellum of mice or PBMC of HD patients and preHD carriers were separated by SDS–PAGE using NuPAGE^®^ Novex Bis-Tris 4–12% gel (Invitrogen, Carlsbad, CA, USA). The resolved proteins were electroblotted onto Immobilon polyvinylidene difluoride membranes (Millipore, Billerica, MA, USA). Membranes were blocked with SuperBloc^®^ blocking buffer (Pierce, Rockford, IL, USA) and incubated with anti-aconitase 2 antibody (1:2000 dilution, Proteintech Group Inc, Chicago, IL, USA), anti-transglutaminase 2 (1:7500 dilution, Abcam, Cambridge, UK), anti-β-actin antibody (1:5000 dilution, BioLegend, San Diego, CA, USA) or anti-Gapdh antibody (1:400 dilution, Santa Cruz, CA, USA) at 4 °C overnight followed by the corresponding secondary antibody for 1 h at room temperature. Immunoreactive bands were detected by enhanced chemiluminescence (ECL, Pierce, Rockford, IL, USA) and recorded using Kodak BioMax light film. The resulting bands were scanned and measured for density using Image Pro software (Image Pro Plus 5.0, Media Cybernetics, MD, USA). Levels of the indicated protein were normalized with an internal control (β-actin or Gapdh).

### 4.5. RNA Isolation and Quantitative Real-Time PCR (QRT-PCR)

Total RNA from striatum of Hdh^(CAG)150^ mice (heterozygous, *n* = 8) and WT littermates (*n* = 8) at 16 months of age was extracted using the Trizol (Invitrogen, Carlsbad, CA, USA), and reverse transcribed into cDNA using SuperScript^TM^ III reverse transcriptase (Invitrogen, Carlsbad, CA, USA). QRT-PCR was performed on a cDNA amount equivalent to 100 ng total RNA using the TaqMan PCR Core Reagent Kit and the Sequence Detection system (ABI Prism 7900, Applied Biosystems, Foster City, CA, USA). Primers (forward primer 5′-CCTAAGGACAGCAGTGGGC-3′, reverse primer 5′-CCCGATCGGACTTTGA-3′) and TaqMan probe (CTCCAGATGCAGACGAGCTT) designed by using Primer Express software, Version 2.0, Applied Biosystems were used for amplification of *Aco2* cDNA. Primers Mm00446968_m1 and TaqMan probe (GGTTAAGGTTGCAAGCTTGCTGGTG) were used in QRT-PCR for *Hprt1.* Relative gene expressions were calculated using the 2^Δ*C*t^ method, Δ*C*_t_ = *C*_t_ (*Hprt1*) − *C*_t_ (*Aco2*), in which *C*_t_ indicates cycle threshold (the fractional cycle number where the fluorescent signal reaches detection threshold). *Hprt1* was used as an internal control. *Aco2* mRNA was measured in duplicate for each sample.

### 4.6. Mitochondria Isolation from PBMC of HD Patients and Brain Tissues of Mice for Aco2 Activities

PBMC isolated from HD patients and the controls were homogenized (GLAS-COL K54 Homogenizer) in HDGC buffer (20 mM HEPES pH 7.4, 0.2 mM PMSF, 1 mM DTT, 10% glycerol, 2 mM sodium citrate, 1X proteinase inhibitor). Tissue of different brain regions from heterozygous (*n* = 6) and homozygous (*n* = 4) Hdh^(CAG)150^ mice at 16 months of age, and from R6/2 mice (*n* = 6) and their littermates (*n* = 6) at 7, 10 and 13 weeks of age were subjected to mitochondrial isolation for Aco2 activity assay. Each tissue sample was homogenized in ice-cold buffer (0.2 mM sodium citrate, 50 mM Tris-HCl, pH 7.4) for 40 s at 210 rpm. The homogenate was centrifuged at 800× *g* for 10 min at 4 °C. The supernatant was transferred into a new tube and centrifuged at 20,000× *g* for 10 min at 4 °C. The resulting pellet was re-suspended in ice-cold 0.2 mM sodium citrate and then sonicated for 20 s. Before the Aco2 assay, the extract was further diluted to 50–500 μg/mL with Assay Buffer.

### 4.7. Assay for Aco2 Activity

The kit BIOXYTECH Aconitase-340™ (OXIS Health Products, Inc., Portland, OR, USA) was used to measure Aco2 activity. Aco2 isomerizes citrate into isocitrate. Isocitrate is catalyzed by isocitrate dehydrogenase to form α-ketoglutarate. The BIOXYTECH Aconitase-340™ Assay was used to measure the simultaneous formation of nicotinamide adenine dinucleotide phosphate (NADPH) from NADP^+^. The formation of NADPH is assessed by the increased absorbance at 340 nm. Under adequate conditions, the rate of NADPH production is reflecting the Aco2 activity. The expression level of Aco2 is expressed in units of activity (mU/mg protein). Aco2 activity was measured in duplicate for each sample and for all samples at the same time. The mean value of two measurements for each sample was used for the analysis.

### 4.8. Statistical Analysis

The Statistical Program for Social Sciences (SPSS) statistical software (version 16.0) was used for the statistical analysis and the data are displayed as means ± standard errors (SE). Differences in rotarod performance were analyzed by two-way ANOVA with post-hoc Tukey test. Differences in western blot analysis, mRNA expression levels, and enzyme activities between animal groups were analyzed by two-tailed Student’s *t*-test or one way ANOVA with post-hoc Tukey test where appropriate. Differences in western blot analysis and enzyme activities between the controls, HD patients and preHD carriers were analyzed by Mann–Whitney–*U* or Kruskal–Wallis test where appropriate. Spearman correlation analysis was applied to evaluate the correlations between the Aco2 activity and UHDRS (motor score, independence scale, and functional capacity), CAG repeat length, disease duration, age or age onset. The values of *p* < 0.05 were considered significant.

## 5. Conclusions

We demonstrate that Aco2 protein and enzyme activity are decreased in the brain of both Hdh^(CAG)150^ and R6/2 mice. NAC has beneficial effect on both rotarod performance and Aco2 activity in striatum of R6/2 HD mice. Finally, we show decreased Aco2 protein level in PBMC of HD patients and decreased Aco2 activity in PBMC of both HD patients and PreHD carriers.

## Figures and Tables

**Table 1:** Clinical characteristics of the Huntington’s disease (HD) patients and the controls.

Parameter	Controls (*n* = 25)	PreHD Carriers (*n* = 6)	HD Patients (*n* = 19)	HD1 Patients (*n* = 7)	HD2 Patients (*n* = 12)
Gender (male/female)	15/10	4/2	13/6	5/2	8/4
Age (years)	48.1 ± 2.4 (30–76)	46.5 ± 7.8 (21–69)	48.7 ± 2.6 (24–67)	51.4 ± 5.5 (24–67)	47.3 ± 2.6 (25–59)
Age at symptom onset (years)			44.4 ± 2.6 (15–62)	43.7 ± 5.9 (15–62)	44.8 ± 2.5 (24–56)
Disease duration (years)			4.5 ± 0.9 (1–15)	7.9 ± 1.7 (3–15)	2.5 ± 0.3 (1–4)
Expanded CAG repeat No		40.2 ± 0.7 (38–43)	44.9 ± 1.3 (40–62)	46.7 ± 2.7 (41–62)	43.8 ± 1.2 (40–56)
UHDRS					
Motor score		0	30.8 ± 4.7 (9–69)	50.3 ± 7.3 (24–69)	19.4 ± 2.9 (9–37)
Independence scale		100	81.1 ± 4.6 (40–100)	60.0 ± 6.5 (40–80)	93.3 ± 1.9 (80–100)
Functional capacity		13	9.9 ± 0.7 (3–13)	6.4 ± 1.0 (3–10)	12.0 ± 0.25 (11–13)

PreHD: pre-symptomatic HD mutation. HD1: more severe disease group. HD2: milder disease group; Values are expressed as means ± SE (range; minimum–maximum); UHDRS: The Unified Huntington’s Disease Rating Scale. Scale ranges (normal to most severe) include motor score (0 to 124), independence score (100 to 10), and functional capacity (13 to 0).

## Supplementary Materials

Supplementary materials can be found at www.mdpi.com/1422-0067/18/11/2480/s1.
