# Supplementary material for: Altered Aconitase 2 Activity in Huntington’s Disease Peripheral Blood Cells and Mouse Model Striatum
Source: Int J Mol Sci. 2017 Nov 21;18(11):2480. doi: 10.3390/ijms18112480 (PMC5713446; doi:10.3390/ijms18112480)
Supplement: Supplementary file 1 [file ijms-18-02480-s001.pdf]

Altered Aconitase 2 Activity in Huntington's Disease Peripheral Blood Cells and Mouse Model Striatum

Chiung-Mei Chen *, Yih-Ru Wu and Kuo-Hsuan Chang

Department of Neurology, Chang Gung Memorial Hospital, Linkou; Chang-Gung University College of Medicine, Taoyuan, 33305, Taiwan; yihruwu@cloud.cgmh.org.tw (Y.-R.W.); gophy5128@gmail.com (K.-H.C.)

* Correspondence: cmchen@adm.cgmh.org.tw; Tel.: +886-3-3281-200 (ext. 8729); Fax: +886-3-3288-7226

Supplementary Materials and Methods

Two-dimensional electrophoresis (2-DE) and imaging analysis

Mouse striatum from Hdh$^{(CAG)150}$ mice (B6.129P2-Hdhtm2Detl/J) (n= 5) or their littermates (n= 5) was ground with liquid nitrogen and lysed with lysis buffer (7 M urea, 2 M thiourea, 4% w/v CHAPS) for protein extraction. The protein was purified by following the manufacture's protocol of ReadyPrep 2-D Cleanup Kit (BioRad, Hercules, CA, USA). Briefly, 100 µL of protein sample was mixed with 300 µL of precipitating agent 1, and incubated on ice for 15 min. 300 µL of precipitating agent 2 was then added to mix, and centrifuged at 12,000 x g for 5 min to form a pellet. After removing the supernatant, the pellet was washed several times and re-suspended with the rehydration buffer (8 M urea, 4% w/v CHAPS, 0.2% w/v Bio-Lyte pH 3-10 and 50 mM DTT).

The isoelectric focusing electrophoresis (IEF) of protein sample from mouse striatum was performed using a commercial 17 cm nonlinear IPG from pH 3 to 10 purchased from BioRad Biosciences (Bio-Rad, Herculus, CA). Analytical loading protein 300 µg was diluted to 330 µL with rehydration buffer. After protein loading, the immobilized pH gradient (IPG) rehydrated strip was covered with mineral oil, and the strip holder was transferred to the focusing unit. IEF was performed as follows: active 50 V for 12 h, 500 V for 1.5 h, 4000 V for 3500 voltage-hours, 8000 V for 65000 voltage-hours. After IEF, IPG strips were placed in an equilibration buffer (6 M urea, 2% SDS, 20% glycerol w/v, 375 mM Tris-HCl, pH 8.8) containing 2% DTT for 20 min at 50 rpm on a rotator shaker. The strips were then transferred to the equilibration solution containing 2.5% iodoacetamide for another 20 min before being proceeded to a vertical 12.5% SDS polyacrylamide gel electrophoresis (SDS-PAGE) for the second dimension separation.

The gels were visualized with SYPRO Ruby staining using a commercially available SYPRO Ruby buffer (Molecular Probes, Eugene, OR). Protein patterns in the gels were scanned using a high-resolution scanner (ProXPRESS 2D Proteomic Imaging System, PerkinElmer). Gel image matching and spot density quantification and normalization were performed using Progenesis software (Progenesis Discovery, Nonlinear Dynamics, Durham, NC). The relative intensities of spots were used for comparison between the two groups and the significantly different spots (1.5-fold increase or decrease) were selected for protein identification.

Protein identification by matrix-assisted laser desorption/ionization time-of-flight mass spectrometry (MALDI-TOF MS) analysis

Differentially expressed protein spots were excised from the gel and washed with 25 mM NH_4HCO_3/50% acetonitrile (pH 8.5) for 15 min several times. After removing washing liquid, acetonitrile was added to cover the gel particles. After drying with a vacuum centrifuge, the gel particles were in-gel digested with freshly prepared trypsin (10 ng trypsin in 5 µl of l 25 mM NH_4HCO_3, pH 8.5) at 37°C for overnight. The resulting peptides were extracted by 50% acetonitrile containing 5% trifluoroacetic acid and analyzed by MALDI-TOF MS.

MALDI-TOF MS analysis was performed using an Ultraflex MALDI-TOF MS instrument (Bruker Daltonics Inc.). Identification of proteins was performed using Mascot software (available at www. matrixscience.com) against all entries in the NCBInr database. A mass deviation of 100 ppm was allowed for inquiry, but the mass accuracy was usually better than 50 ppm. Coverage of the full-length protein exceeding 25% was considered to be sufficient unless there were some obvious

conflicts of molecular weight or isoelectric point between spots and identified proteins. Matching peptides with one missed cleavage were considered as adequacy.

Detection of intracellular aggregates of R6/2 mice

Brain sections (20 μm) of R6/2 mice (n=4) with and without NAC treatment and littermates with saline treatment (n=4) were subjected to immunohistochemical analyses of intracellular aggregates. Cells harboring aggregates of mutant huntingtin (Htt) was quantitated in a blinded fashion. Single-antigen immunostaining will be carried out using the avidin–biotin–peroxidase complex (ABC) method. We used EM48 (anti-huntingtin) at 1:2000 dilution to stain intracellular aggregates. Four different consecutive sections of each mouse brain were labeled with EM48 and aggregates within an area of 87x10^{3}μm^2 of each section were quantified. The mean of aggregate counts of four brain sections from each mouse was used for statistical analysis.

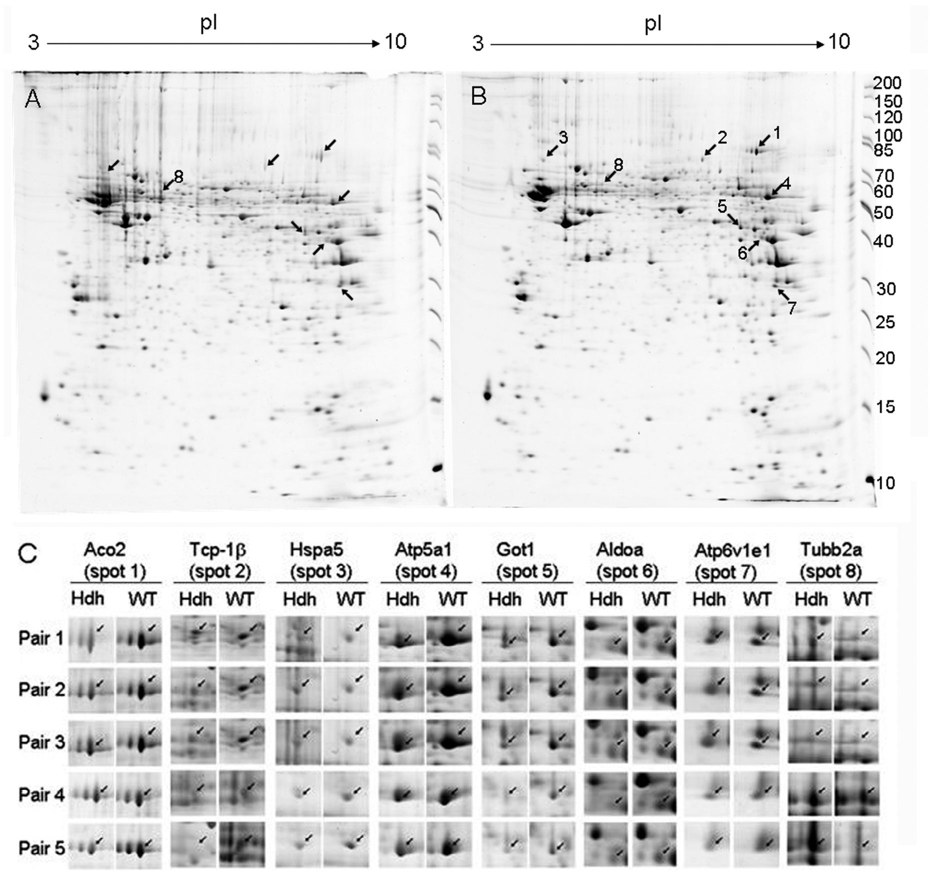

Figure S1. The representative 2-DE map of paired striatal proteome comparisons between heterozygous Hdh$^{(CAG)150}$ (A) and the wild-type mice (B) at 16 months of age. Spots 1~7 (B) were down-regulated and spot 8 (A) was up-regulated in Hdh$^{(CAG)150}$ mice. (C) The cropped images of differentially-expressed proteins (spots 1-7 as indicated in B and spot 8 in A) from 2-DE maps of 5 striatal pairs of Hdh$^{(CAG)150}$ (Hdh) and wild-type (WT) mice at 16 months of age.

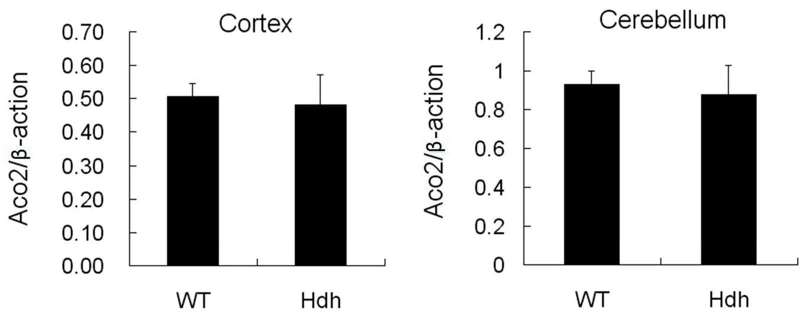

Figure S2. Aconitase (Aco2) protein expression in the cortex and cerebellum of Hdh$^{(CAG)150}$ (Hdh) (n = 4) compared with their wild type (WT) littermates (n = 4) at 16 months of age. Data are presented as means ± SE (standard error bars).

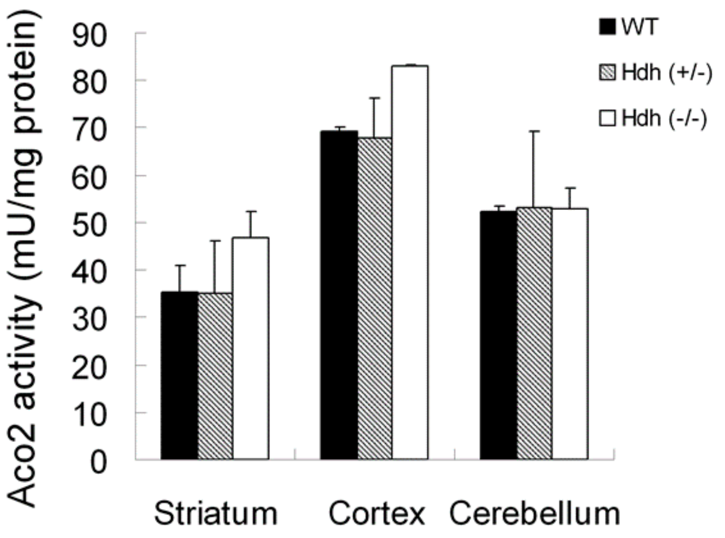

Figure S3. Aco2 activities in the striatum, cortex and cerebellum of heterozygous (n = 6) and homozygous Hdh$^{(CAG)150}$ (n = 4) mice compared to their littermates (n = 6) at 13 months of age. Data are presented as means ± SE (standard error bars).

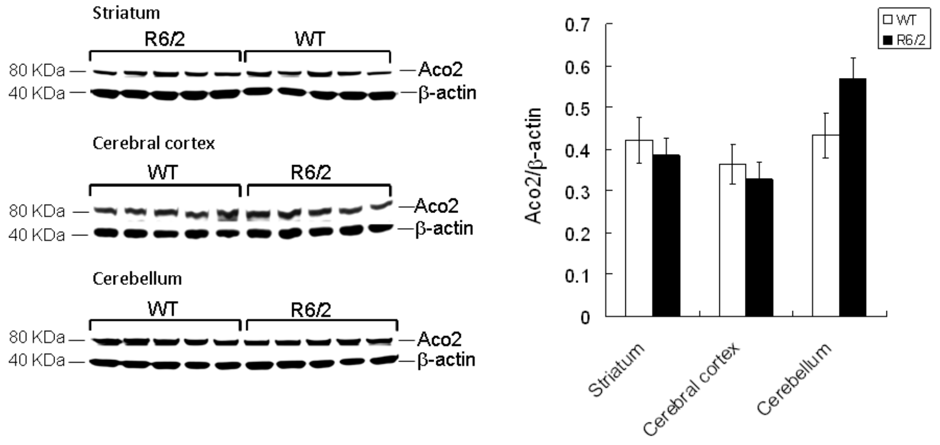

Figure S4. Aco2 protein expression in the brain regions of R6/2 mice. Aco2 expression levels, analyzed by western blot, in the striatum, cerebral cortex, and cerebellum of R6/2 mice (n = 5) were compared with their wild type (WT) littermates (n = 5) at 10 weeks of age. Data are presented as means ± SE (standard error bars).

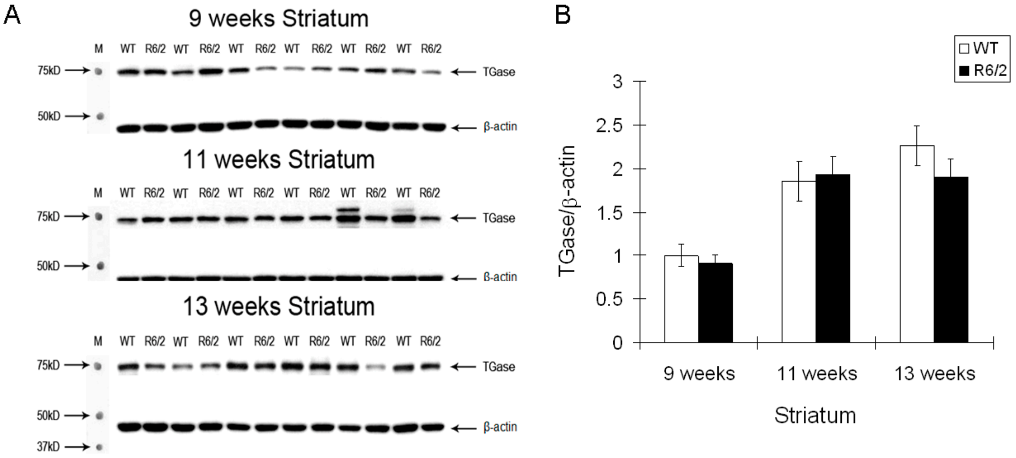

Figure S5. Transglutaminase 2 expression in the striatum of R6/2 mice. Transglutaminase 2 (TGase) expression levels, analyzed by western blot (A), in the striatum of R6/2 mice (n = 6) were compared with their wild type (WT) littermates (n = 6) at 9, 11, and 13 weeks of age (B). The expression levels of Transglutaminase 2 were normalized to that β-actin. M, marker for protein molecular weight. Data are presented as means ± SE (standard error bars).

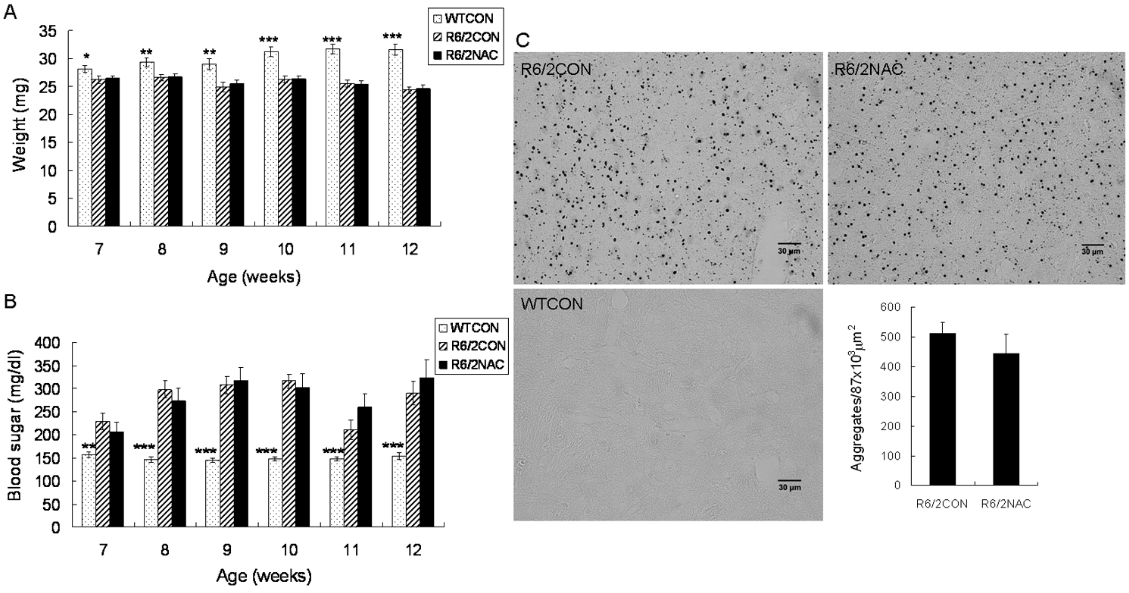

Figure S6. Effects of N-acetyl-l-cysteine (NAC) treatment on body weight, blood sugar, and aggregates in the striatum of R6/2 mice. (A) Body weight and (B) blood sugar of R6/2 mice intraperitoneally injected with NAC (R6/2NAC, n = 15) at 7, 8, 9, 10, 11, and 12 weeks of age were compared with those of R6/2 mice treated with saline (R6/2CON, n = 15). (C) Aggregates in striatum of R6/2 mice intraperitoneally injected with NAC (R6/2NAC, n = 4) were compared with those of R6/2 mice treated with saline (R6/2CON, n = 4) sacrificed at 12 weeks of age. Data are presented as means ± SE (standard error bars). **P* < 0.05, ***P* < 0.01,*** *P* < 0.001, comparisons between WTCON and R6/2NAC, or between WTCON and R6/2CON, one way ANOVA with post-hoc Tukey test.

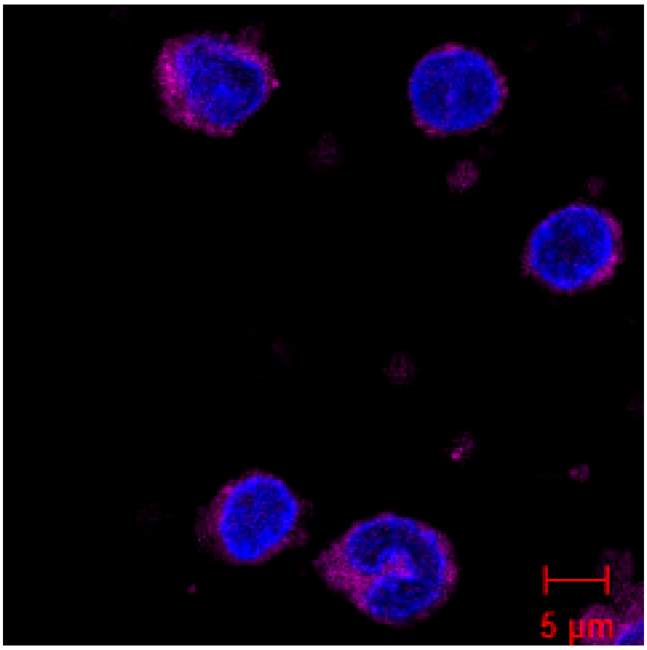

Figure S7. The representative PBMC isolated from peripheral blood of a participant. PBMC was subjected to immunocytochemical staining using anti-CD1b antibody (1:200 dilution, Abcam) (red) with nuclei being stained by Hoechst (blue).

Table S1. 2-DE and MALDI-TOF MS identification of proteins that were differentially expressed in the striatum between heterozygous Hdh$^{(CAG)150}$ mice and their littermates at 16 months of age.

**Spot** [a]	**Accession No.**	**Protein name**	**pI/M.W.**	**Score** [b]	**Coverage** [c] **(%)**	**Average fold change (Hdh$^{(CAG)150}$/WT)**[d]	**Frequency** [e], ***p***
		Down-regulated in Hdh$^{(CAG)150}$ mice					
1	gi|18079339	Aconitase 2 (Aco2)	8.08/85410	65	18	-1.9	4/5, $p < 0.01$
2	gi|6671700	T-complex protein 1 subunit beta (Tcp-1β)	5.97/57411	66	29	-1.6	3/5, $p < 0.05$
3	P20029	Heat shock 70 kDa protein 5 (Hspa5)	5.07/72377	88	25	-1.4	3/5, $p < 0.05$
4	Q03265	ATP synthase subunit alpha, mitochondrial precursor (Atp5a1)	9.22/59716	64	23	-2.9	4/5, $p < 0.01$
5	gi|6754034	Aspartate aminotransferase, cytoplasmic (Got1)	6.68/46202	114	35	-1.7	3/5, $p < 0.05$
6	gi|6671539	Aldolase 1, A isoform (Aldoa)	8.31/39331	88	43	-1.5	3/5, $p < 0.05$
7	gi|45504359	Vacuolar H+ ATPase E1 (Atp6v1e1)	8.44/26141	100	50	-2.2	4/5, $p < 0.05$
		Up-regulated in Hdh$^{(CAG)150}$ mice					
8	gi|34740335	Tubulin alpha-2 (Tubb2a)	4.94/50120	77	38	1.8	3/5, $p = 0.08$

a. Spots numbered as shown in Supplementary Figure; b. The MASCOT search score of identified proteins; c. The percentage of sequence coverage of matched peptides in the identified protein; d. The average ratio of protein expression levels between 5 Hdh$^{(CAG)150}$ and the 5 wild-type littermate (WT) mice; e. The frequency of the target protein for which the expression ratio of Hdh$^{(CAG)150}$/WT was >1.5, or < -1.5 based on the Progenesis software analysis on the 2-DE gels of the paired CSF specimens. $P < 0.05$ indicates a significant difference in expression levels between Hdh$^{(CAG)150}$ and WT mice analyzed by paired student's *t*-test.
